# Maternal socio-demographic and psychological predictors for risk of developmental delays among young children in Mongolia

**DOI:** 10.1186/s12887-018-1017-y

**Published:** 2018-02-19

**Authors:** Amarjargal Dagvadorj, Duurenbayar Ganbaatar, Olukunmi O. Balogun, Naohiro Yonemoto, Bayasgalantai Bavuusuren, Kenji Takehara, Rintaro Mori, Moe Akahira-Azuma

**Affiliations:** 1 Department of Health Policy, National Center for Child Health and Development, 2-10-1 Okura, Setagaya-ku, Tokyo, 157-8535 Japan; 2 Clinical Epidemiology Program, Ottawa Hospital Research Institute, Ottawa, Canada; 3 Department of Pediatrics, Mongolian National University of Medical Science, Ulaanbaatar, Mongolia; 4 Department of Biostatistics, School of Public Health, Kyoto University, Kyoto, Japan; 5 Department of Pediatrics, National Center for Global Health and Medicine, Tokyo, Japan

**Keywords:** Child development, Developing country, Maternal education, Mongolia, Risk of child development

## Abstract

**Background:**

Factors influencing child development are not well studied in developing settings, and especially in Mongolia. This cohort study examined the relationship between maternal socio-demographic and psychological conditions on risk of young child developmental delay.

**Methods:**

A total of 150 children aged between 13 ~ 24 months old participated in this study. The participants were randomly selected from a pre-existing cohort of 1297 children who were involved in a study on infant bilirubin nomogram development conducted at a tertiary health facility in Mongolia between 2012 and 2013. Child development was evaluated using the Mongolian Rapid Baby Scale (MORBAS), a validated scale for child development. The potential factors for child developmental delay were assessed using a pre-tested questionnaire comprising of 52 questions. Fisher’s exact test and multivariable logistic regression analysis were conducted.

**Results:**

Seventeen (11%) out of the 150 children that participated in the study were at risk of developmental delay. There was a negative association between the risk of child developmental delay and higher maternal education (AOR 0.15, 95% CI: 0.03–0.66). Increasing maternal age (AOR 1.12, 95%CI: 0.98–1.27), maternal depression symptoms (AOR 4.93, 95%CI: 0.93–26.10), child gender being female (AOR 0.25, 95%CI: 0.06–1.00) and being from single mother household (AOR 0.14, 95%CI: 0.01–1.11) were also predictors for risk of developmental delay – although the association was marginal.

**Conclusions:**

Our findings suggest that being of underprivileged social status, and poor psychological condition of mothers in Mongolia possibly increases the risk of child developmental delays. Interventions targeting these modifiable predictors are needed to develop prevention strategies for child developmental delay.

## Background

Many children in developing countries are exposed to multiple risks which limit their cognitive, motor, and social-emotional development, and research on factors affecting early child development is scarce in many of these countries. An estimated 20–25% of young children in developing countries are known to be experiencing lack of basic needs their normal development such as lack of cognitive stimulation, inadequate nutrition resulting in stunting, iron and iodine deficiency [1]. Various factors contribute to the risk of child developmental delays and can be broardly divided into biological and psychosocial factors [1].

The main biological risk factors that compromise child development include preterm birth [2] and low birthweight (LBW) [1, 3]. As opposed to term babies, lower gestational age at birth babies are reported to have increased likelihood of poorer neurodevelopmental outcomes, especially among infants in low– and middle–income countries [2]. Similarly, being born LBW is known to delay the developmental processes as compared to normal birthweight infants [3]. Specifically, LBW infants are known to have significantly lower mental development and psychomotor development index scores when compared to average birthweight infants [3].

Other biological risk factors include low apgar score and neonatal jaundice. Newborns who have lower apgar scores are reported to be at risk of encephalopathy and other developmental problems and they have adverse outcomes related to asphyxia [4]. A recent review involving studies from both developing and developed countries reported increased risk of developmental delays in children with severe neonatal jaundice [5]. Furthermore, late diagnosis of neonatal jaundice may also cause physical and mental retardation including hearing problems, and visual impairments [5, 6]. These conditions are reported to occur in up to 40% of newborns with moderate hyperbilirubinemia (serum bilirubin concentration 10–20 mg/dl) increasing the risk of other neurodevelopmental problems [7].

Psychosocial risk factors known to hinder child development include maternal depression and child rearing dimensions [8]. Maternal depression may affect child rearing behaviour [9], wherein depressed mothers are more likely to be uninvolved, less sensitive, and negative when interacting with their children [9]. Meanwhile, interventional studies conducted in developing countries showed that cognitive stimulation of young children by their parents promoted higher cognitive functioning in young children significantly [1].

Recent nationwide surveys in Mongolia show that 22.9% of children aged between 2 and 9 years old had mental or physical impairments [10]. However, prevalence of developmental delay and factors that could be putting young children aged between 1 and 2 at risk of developmental delay is not well studied in Mongolia. This area of research is important, as the younger the child, the faster the improvement following an intervention [11]. Reliable and updated information on specific conditions relating to child development would allow us to raise awareness on prevention and intervention strategies for preventing developmental delay in Mongolia. Given that our study was conducted in a developing country, the main conceptual framework is based on the framework proposed by the Walker et al. on child development risk factors in developing countries [1]. This framework includes only modifiable risk factors which can be influenced through interventions or public policy.

Therefore in this study, we aimed to evaluate child development using a validated, country specific scale and determine related predictors in children aged between 13 ~ 24 months old in Mongolia.

## Methods

### Study population

This is a follow-up study to Akahira-Azuma et al., 2015 that established the hour-specific transcutaenus bilirubin nomogram in Mongolian neonates. Study participants were randomly selected from among 1297 healthy term and late-preterm neonates who participated in the baseline study. All infants born to women enrolled at a tertiary level health facility in Mongolia between October 2012 and September 2013 participated in the baseline study. Details of the setting and participants characteristics have been described previously [12]. This follow-up study was conducted among children aged 13–24 months old following telephone recruitment between October and November, 2014. A total of 150 children were recruited.

### Exposure and outcome

According to the framework used in this study [1], risk factors on child development in developing countries can be grouped into four main domains including poverty, socio-cultural factors, biological factors and psychosocial factors. Based on this framework, we made effort to include the main factors from each of the different domains. Poverty level was described by wealth index while for socio-cultural risk factors, we included child gender and maternal education to reflect gender inequity and low maternal education. Biological risks factors included variables representing prenatal and postnatal growth such as delivery mode, gestational age at birth, birthweight, apgar score, transcutaenous bilirubin level, season of birth and exclusive breastfeeding. Biological factors specific to the mother were parity, history of miscarriage, and disease during pregnancy. For psychosocial risks factors, we included environmental and parenting factors such as family crowding, maternal work, single mother household and maternal depression symptoms. Impairment in child development was thereafter assessed in seven child developmental domains.

Baseline characteristics of participants were obtained from the previous study while information regarding the exposures and outcomes were collected during the follow-up survey. The primary outcome in this study was child development. Child development was evaluated using the Mongolian Rapid Baby Scale (MORBAS) [13]. MORBAS is an easy to use rapid screening tool for healthcare providers and parents to evaluate risk of developmental delay in young children. The tool is comprised of seven developmental domains on gross motor, fine motor, cognitive, expressive language, receptive language, social-emotional, and adaptive-behavior. A validation study conducted by comparing the MORBAS with an international gold standard, Bayley-III showed good concurrent, face and content validity [13]. In current study, MORBAS was utilized by healthcare providers at the study hospital to assess risk of developmental delay. Each assessment lasted approximately 15 min.

Information on date of birth, gender, gestational age at birth, birthweight, Apgar score, mode of delivery, maternal age, number of pregnancies, number of deliveries, maternal blood type, jaundice in siblings, feeding at discharge and transcutaenus bilirubin measurement during the first 6 days after birth were obtained from the baseline data [12].

Additional data were collected in the follow-up study using a 52 item pre-tested questionnaire. The questionnaire was divided into two parts and assessed maternal characteristics in terms of socioeconomic status and health (health behavior, history of neonatal jaundice, breastfeeding practices, and disease history) specific to the index child. Information on maternal depression symptoms was obtained using the Self-Reporting Questionnaire (SRQ)-20 [14]. The SRQ-20 has previously been validated among Mongolian women of childbearing age [14] with a cut-off for detecting maternal depression symptoms defined as SRQ-20 score ≥9. Economic status was assessed using data on asset ownership and household characteristics at follow-up and a wealth index was constructed from the data using principal components analysis [15]. Participants were then categorized using this index scores into tertiles according to a 3-point scale ranging from 1 (poor) to 3 (rich).

Given that minimum maternal education attained in our sample was 8 years, maternal education was categorized into two – middle (8–12 years of formal education) and upper (more than 13 years of formal education) levels. Apgar score at 1-min cut-off value was set at 8. Newborns with 1-min Apgar score value lower than 8 were considered to have increased risk of morbidity [16]. Infants were considered small-for-gestational age based on the Mongolian neonate’s values derived from a secondary analysis of the World Health Organization multi-country survey on maternal and newborn health [17]. Seasonal variation at birth is reported to influence hyperbilirubinemia due to the fact that exposure to daylight decreases the level of bilirubin [18, 19]. Given that children’s exposure to daylight dramatically decreases during winter months in Mongolia, we took seasonal variation of birth into account categorizing the season into summer time (from April to September) and winter time (from October to March) [20]. Date of birth provided information regarding seasonal variation at birth.

### Statistical analysis

The sample size for this study was estimated using formula below [20] and assuming a MORBAS score standard deviation of 3.0 [21], with significance level of α = 0.05 using two-sided test with power of 80% (β = 0.20) and an effect size of d = 1.5 [13].

$$ \mathrm{n}=2{\mathrm{SD}}^2{\left({\mathrm{Z}}_{\alpha /2}+{\mathrm{Z}}_{\upbeta}\right)}^2/{\mathrm{d}}^2 $$

Under these assumptions, a total sample size of *n* = 128 was required to detect differences in the risk of developmental delay. Our final sample size was 150 children anticipating a 15% attrition rate. We randomly selected mother/child dyads from the baseline sample of 1297 women. Telephone calls were made to randomly selected numbers. Of the 1297 eligible women, 344 women could not be reached on phone due to connection error. Additionally, phone numbers provided by 248 women were no longer in use and 53 women declined to participate in the study before we reached the desired sample size.

We used Fisher’s exact test and t-test to determine basic characteristics of study participant on categorical and continuous variables respectively. Multivariable logistic regression analysis was used to find the association between predictor variables and the risk of child developmental delay. Predictors included in the analysis were child gender, maternal age, wealth index, crowding, type of household, maternal education, maternal work, history of miscarriage, disease during pregnancy, maternal depression symptoms, and exclusive breastfeeding. Effect sizes are presented as adjusted odds ratios (AOR) with corresponding confidence intervals (CI).

All analyses were conducted using de-identified data in Stata version 13.0 (StataCorp LP, College Station, Texas, USA).

## Results

A total of 150 Mongolian children participated in this study. Basic characteristic of the study children at birth and at baseline are presented in Table 1 and Table 2 respectively. Seventeen (11%) of the 150 children were at risk of developmental delay for at least one of the developmental domains. Specifically, children were at risk of developmental delay in the following domains: gross motor (*n* = 1), expressive language (*n* = 9), receptive language (*n* = 2). One child each was at risk of developmental delay in the other domains except cognitive delay for which no child was at risk.

**Table 1:** Characteristics of child and mother at birth *n* = 150

Baseline data	Category	Distribution		No risk of delay		Risk of delay		*p* value
		n	%	n	%	n	%	
Sex	Male	85	56.7	72	54.1	13	76.4	0.118
	Female	65	43.3	61	45.8	4	23.5	
Maternal age	17–20	4	2.7	3	2.2	1	5.8	0.502
	21–30	81	54	73	54.8	8	47.1	
	≧31	65	43.3	57	42.8	8	47.1	
Maternal age mean(SD)	Year	29.8 (5.2)		29.8 (5.2)		30.2 (5.7)		0.111
Delivery mode	Vaginal	91	60.7	79	59.4	12	70.5	0.439
	Cesarean	59	39.3	54	40.6	5	29.4	
Birthweight mean(SD)	Gram	3592 (446)		3611 (448)		3441 (412)		0.126
Gestational week mean(SD)	Week	38.9 (0.9)		39.0 (0.9)		38.5 (1.3)		0.111
Preterm	≧38 week	143	95.3	128	96.2	15	88.2	0.181
	≦37 week	7	4.7	5	3.7	2	11.7	
Small for gestational age	Yes	4	2.7	4	3	0	0	1
	No	146	97.3	129	96.9	17	100	
Apgar 1 min	≧8	55	36.7	51	38.3	4	23.5	0.292
	≦7	95	63.3	82	61.6	13	76.4	
Apgar 1 min mean (SD)	Score	7.3 (0.6)		7.3(0.6)		7.2(0.5)		0.51
Hyperbilirubinemia	< 10 mg/dL	45	30	38	28.5	7	41.2	0.399
	> 10 mg/dL	105	70	95	71.4	10	58.8	
Season of birth	October–March	73	48.7	63	47.3	10	58.8	0.445
	April–September	77	51.3	70	52.6	7	41.2	
Parity	Primiparous	48	32	42	31.5	6	35.3	0.786
	Parous	102	68	91	68.4	11	64.7

**Table 2:** Characteristics of child and mother at birth (13–24 months) n = 150

Follow-up data	Category	Distribution		No risk of delay		Risk of delay		*p* value
		n	%	n	%	n	%	
Mothers								
Wealth index	Poor	51	34.2	44	33.3	7	41.2	0.852
	Middle	49	32.9	44	33.3	5	29.4	
	Rich	49	32.9	44	33.3	5	29.4	
Crowding	< 5	96	64.4	86	65.2	10	58.8	
	≧5	53	35.6	46	34.9	7	41.2	
Single mother household	Yes	8	5.3	5	3.8	3	17.7	0.048
	No	142	94.7	128	96.2	14	82.4	
Maternal education	Middle	16	10.7	11	8.3	5	29.4	0.021
	Upper	134	89.3	122	91.7	12	70.6	
Maternal work	Yes	75	50.0	68	51.1	7	41.2	0.608
	No	75	50.0	65	48.9	10	58.8	
History of miscarriage	Yes	37	24.7	34	25.6	3	17.6	0.566
	No	113	75.3	99	74.4	14	82.4	
Alcohol use during pregnancy	Yes	12	8.0	11	8.3	1	5.9	1.000
	No	138	92.0	122	91.7	16	94.1	
Smoking during pregnancy	Yes	6	4.0	5	3.8	1	5.9	0.52
	No	144	96.0	128	96.2	16	94.1	
Disease during pregnancy	Yes	81	54.0	75	56.4	6	35.3	0.124
	No	69	46.0	58	43.6	11	64.7	
Maternal depression symptoms	Yes (> 9)	20	13.3	17	12.8	3	17.7	0.703
	No (< 8)	130	86.7	116	87.2	14	82.4	
Children								
Exclusive breastfeeding	< 6 month	71	47.3	63	47.4	8	47.1	1.000
	≧6 month	79	52.7	70	52.6	9	52.9	
Developmental delays in relatives	Yes	11	7.3	10	7.5	1	5.9	1.000
	no	139	92.7	123	92.5	16	94.1	

Among our study participants, 7 (4.7%) children were born preterm, 95 (63.3%) were scored < 7 for 1 min Apgar score, and 105 (70%) children had moderate hyperbilirubinemia (transcutaneous bilirubin level of > 10 mg/dl) at birth (Table 1).

Eight (5.3%) children were from single mother households while 16 (10.6%) children were born to mothers educated below middle school educational level (less than 12 years of formal education). Alcohol and tobacco use during pregnancy was reported by 12 (8%) and 6 (4%) mothers respectively. Twenty (13.3%) mothers had depression symptoms as detected by the SRQ-20 test.

Two multivariable analysis models were constructed to determine predictors of developmental delay at baseline and at follow-up. In the first model, we found no clear association between baseline factors and the risk of developmental delay (Table 3). Further, we constructed another model using the follow-up predictors. We found that higher maternal education was an important protective factor against risk of developmental delay (AOR-0.15; 95%CI [0.03–0.66]). Other predictors associated with risk of developmental delays were gender being female (AOR- 0.25; 95% CI [0.06–1.00]); and increasing maternal age (AOR- 1.12; 95%CI [0.98–1.27]). Additional predictors for risk of developmental delay in the infants were maternal depression symptoms (AOR- 4.93; 95%CI [0.93–26.10]) and being a single mother (AOR-0.14; 95%CI [0.01–1.11]) although the associations were only marginal (Table 4).

**Table 3:** A model of predictors of developmental delay at baseline n = 150

Predictors	Category	OR	95%CI	*p* value	AOR	95%CI	*p* value
Child sex	Male	1			1		
	Female	0.36	0.11–1.17	0.09	0.32	0.09–1.15	0.082
Maternal age	Per 1 year	1.02	0.92–1.12	0.705	1.03	0.91–1.16	0.604
Delivery mode	Vaginal	1			1		
	Cesarean	0.78	0.45–1.35	0.377	0.61	0.18–2.01	0.410
Gestational week	Per 1 week	0.58	0.35–0.97	0.038	0.63	0.35–1.12	0.116
Birthweight	Per 100 g	0.91	0.80–1.03	0.14	0.96	0.83–1.11	0.629
Apgar score at 1 min	Per score 1	0.8	0.38–1.70	0.24	0.83	0.36–1.92	0.671
Hyperbilirubinemia	< 10 mg/dL	1			1		
	> 10 mg/dL	0.57	0.20–1.61	0.29	0.53	0.16–1.66	0.278
Season of birth	October–March	1			1		
	April–September	0.63	0.22–1.75	0.377	0.51	0.17–1.54	0.237
Parity	Primiparous	1			1		
	Parous	0.84	0.29–2.44	0.757	1.01	0.26–3.72	0.998

Multiple logistic regression analysis adjusted for variables in this table

**Table 4:** A model of predictors of developmental delay at follow-up *n* = 149

Predictors	Category	OR	95%CI	p value	AOR	95%CI	*p* value
Child sex	Male	1			1		
	Female	0.36	0.11–1.17	0.09	0.25	0.06–1.00	0.050
Maternal age	Per year	1.02	0.93–1.12	0.642	1.12	0.98–1.27	0.073
Wealth index	Rich	1			1		
	Poor	0.83	0.45–1.56	0.578	1.37	0.29–6.45	0.687
	Middle				0.62	0.14–2.74	0.536
Crowding	< 5	1			1		
	≧5	1.3	0.46–3.66	0.609	0.68	0.19–2.45	0.565
Single mother household	Yes	1			1		
	No	0.18	0.03–0.84	0.03	0.14	0.01–1.11	0.063
Maternal education	Middle	1			1		
	Upper	0.21	0.06–0.72	0.013	0.15	0.03–0.66	0.012
Maternal work	No	1			1		
	Yes	0.66	0.24–1.86	0.442	0.51	0.15–1.68	0.272
History of miscarriage	Yes	1			1		
	No	1.6	0.43–5.91	0.479	1.81	0.39–8.22	0.441
Disease during pregnancy	Yes	1			1		
	No	2.37	0.82–6.78	0.108	2.61	0.67–10.15	0.165
Maternal depression symptoms	No (< 8)	1			1		
	Yes (> 9)	1.46	0.38–5.62	0.58	4.93	0.93–26.10	0.060
Exclusive breastfeeding	< 6 month	1			1		
	≧ 6 month	1.01	0.36–2.78	0.981	1.42	0.40–5.04	0.579

Multiple logistic regression analysis adjusted for variables in this table

## Discussion

The purpose of the current study was to examine major biological and psychosocial factors associated with the risk of young child developmental delay. Eleven percent of the study children were at risk of developmental delay while higher maternal educational level was found to be strongly protective against the risk of child development delay. Although the risk of child developmental delay increased due to other modifiable predictors such as belonging to single mother households and the presence of depression symptoms in mothers, these associations were modest.

The findings from the current study showed that children of less educated mothers were at increased risk of developmental delay compared to children of more educated mothers. Studies from other countries also support our findings [22, 23]. Maternal education has been shown to be associated with many positive aspects of the child development throughout their growth [22]. Moreover, prior studies show that highly educated mothers in developing countries are more likely to seek appropriate care for their children [23]. For example, findings from studies examining the association between increases in maternal education and children’s school readiness show that expressive and receptive language ability, and cognitive test results were better among pre-school children who had mothers with higher education [24]. Similarly, mothers with at least 2 years of college education were shown to have fluent vocabulary and supportive style of child directed speech, which correlated to richer vocabulary of children when compared to less educated mothers [25]. Given that women’s education directly impacts on their autonomy, educated mothers may be more likely to make decision concerning their children’s health condition independently, through perceiving that their behavior is their own responsibility [26].

In our study, maternal depression symptoms was found to be associated with risk of developmental delay although the association was modest. Notwithstanding, this finding is important given that child rearing practices could be affected by maternal depression [9]. Prior studies have shown that depressed mothers are more negatively involved with their children’s daily activities [27] resulting in short and long term consequences. Recently, maternal depression early in a child’s life is reported to be a risk factor for low math score during adolescence [28]. Therefore, screening and implementing preventive interventions for maternal depression may enhance not only improvement in young child development, but also influence school achievement during adolescence. For example, early parenting-programs are reported to improve parental responses [29–31] and lead to increase in the abilities of depressed mothers to support their child’s executive functions which controls and regulates a child’s thoughts and behaviors [32, 33].

Furthermore, belonging to a single-mother household was weakly associated with risk of child developmental delay. Prior research shows that transition to single-parent household reduces the child’s well-being, because it can bring emotional and economic losses to children [34]. Particularly, those who experienced parent’s divorce in their earlier pre-school age are reported to have adaptive-behavior problems [34]. In the long-term, children growing up in father-absent households were shown to be more likely to suffer from adolescent social problems, such as school dropout, substance abuse, and juvenile delinquency [35]. This might be because single mothers go through more stress and have higher risk of mental distress than undivorced women [36]. However, children with behavior problems who lived in supportive environments have been shown to have greater chances for advanced development of self-regulatory skills than children who lived in unsupportive family settings [34]. This emphasizes the importance of family member’s support, and careful attention toward children’s conduct to ensure child self-regulation as it is an essential factor for school readiness.

To the best of our knowledge, this is the first study conducted in Mongolia assessing maternal socio-demographic and psychological predictors related to young child development in Mongolia. We also examined the influence of hyperbilirubinemia on child development using the first country-specific validated screening tool to assess child development. Although the study did not include all the children from the baseline study, our study population was shown to be representative when we compared the characteristic of the study population to the rest of the cohort that did not participate in this study.

Our study has findings with important implications for child development, however, there are several limitations to this study. First, factors that change with family situation such as home environment, changing economic status, cognitive stimulation in the home, and family structure change could not be included in our study. Although we controlled for factors such as type of household i.e. being single mother household or not, such home environment factors may not have been fully accounted for. Second, other potentially relevant variables such as reduced access to services, nutrient deficiencies, environmental toxins, and maternal exposure to violence were not considered. Third, the health condition of the children, including history of jaundice was obtained 1–2 years after birth thus introducing the possibility of recall bias. Fourth, results may not be generalizable to the whole population as the data used in this study is from a cohort of infants born within a single hospital. However, institutional delivery is universal in Mongolia and our study location was a tertiary level national center with more than 10,000 deliveries per annum. Additionally, being a tertiary level care center, mothers come from all over the country for delivery in our study location hospital. Lastly, although the sample size was calculated based on robust statistical methods, we included only a small sample of participants in the study which may have hampered more sophisticated analyses. Given that mothers were randomly selected into this study, we think that our findings may reflect a true situation.

All the predictors described in current study are modifiable, which suggests that women empowerment through education can bring benefit thus maximizing child potential. Maternal education is not only a tool to emerge from poverty, but it also has the capacity to prevent loss of human potential [1].

## Conclusions

In conclusion, we found that maternal education plays an important role in reducing the risk of child developmental delay. Furthermore, psychosocial factors such as being from single-mother household and maternal depression symptoms are associated with risk of child developmental delay although this finding was modest. These results suggest that underprivileged social and psychological conditions of mothers in Mongolia contribute to risk of child developmental delays. Thus, our study findings provide valuable contribution towards making appropriate policies, preventive measures and intervention that could be directed toward vulnerable mothers who are less educated, single and have risk of psychological disorder.

Finally, our study emphasizes the need for future studies to highlight the contribution of developmental psychology on the risk of child development.

## Abbreviation

AOR: Adjusted Odds Ratio
CI: Confidence Interval
LBW: Low birthweight
MORBAS: Mongolian Rapid Baby Scale
SRQ: Self-Reporting Questionnaire
